# Exploring protective associations between the use of classic psychedelics and cocaine use disorder: a population-based survey study

**DOI:** 10.1038/s41598-022-06580-2

**Published:** 2022-02-16

**Authors:** Grant M. Jones, Matthew K. Nock

**Affiliations:** Department of Psychology, Harvard University, 33 Kirkland St, Cambridge, MA 02138 USA

**Keywords:** Psychology, Human behaviour

## Abstract

Cocaine Use Disorder (CUD) is a significant public health problem associated with elevated morbidity and mortality within the United States. Current behavioral treatments have limited efficacy and there are currently no FDA approved pharmacological treatments for CUD. Classic psychedelics might be associated with lowered odds of substance misuse and may effectively treat various forms of addiction. Thus, the goal of this study is to assess protective associations that lifetime use of classic psychedelics may share with CUD within a nationally representative sample of the U.S. We used data from The National Survey on Drug Use and Health (NSDUH) (2015–2019) and conducted survey-weighted multivariable logistic regression to test whether each of four classic psychedelics (peyote, mescaline, psilocybin, LSD) conferred lowered odds of CUD and its related 11 sub-criteria. Participants were 214,505 adults in the NSDUH (2015–2019) aged 18 and older. Peyote conferred lowered odds of CUD, reducing the odds of CUD by over 50% (aOR: 0.47). All other substances (including other classic psychedelics) either shared no association to CUD or conferred increased odds of CUD. Furthermore, sensitivity analyses revealed peyote to confer sharply lowered odds of the majority (seven of 11) of CUD criteria as well (aOR range: 0.26–0.47). Peyote use is associated with lowered odds of CUD. Future inquiries into third variable factors (i.e., demographic/personality profiles of individuals who use peyote, motivational/contextual factors surrounding peyote use) that may underlie our observed associations may reveal protective factors that can inform treatment development for CUD. Additionally, future longitudinal studies can shed further light on whether there is a temporal link between peyote use and lowered odds of CUD.

## Introduction

Cocaine use disorder (CUD) is a major public health problem afflicting approximately one million people within the United States^1^. Furthermore, CUD has a significant morbidity and mortality burden, as the disorder sharply raises one’s risk for mental illness, violent crime, vascular-related illnesses like stroke and heart attack, and overdose death^2–9^. Although behavioral interventions for CUD demonstrate effectiveness^10^, there are many with substance use disorders who are not supported by behavioral interventions^11^. Furthermore, there are currently no FDA-approved pharmacological interventions for CUD, and researchers have noted the need for innovation and high risk/high reward inquiries into potential pharmacological treatments for CUD^12,13^. Accordingly, there is also a need to better understand potential protective factors for CUD as these may inform novel effective treatment approaches as well^14^.

Classic psychedelics might be potential treatments for CUD; and, better understanding the population of individuals who use classic psychedelics may allow us to identify protective factors for CUD. Classic psychedelics are hallucinogens that are naturally occurring or derived and give rise to marked perceptual shifts, such as time dilation, visual and auditory hallucinations, and profound spiritual experiences. This class of hallucinogens is known to confer their effects by acting as serotonin 2A agonists. Some of the most commonly used classic psychedelics are psilocybin (the active compound in “magic mushrooms”), peyote (*Lophophora williamsii*—a cactus containing alkaloids with psychoactive properties), mescaline (the primary psychoactive compound in peyote), and LSD (synthesized from the Ergot fungus). Classic psychedelics have been used by indigenous cultures for thousands of years as sacrament within ceremony and ritual as well^15^.

Research has linked classic psychedelics to the alleviation of substance use disorders^16^. Meta-analyses and reviews of historical trials have suggested LSD may alleviate alcohol use disorder^17,18^, although many of these historical trials lacked sufficient power and reported equivocal findings^16^. However, more recent studies link classic psychedelics to reductions in substance use disorder as well. First, Johnson et al. conducted an open label pilot study with 15 nicotine-dependent smokers and found psilocybin to elicit abstinence in 80% of participants that was sustained at 6-month follow-up^19^. Second, a proof-of-concept trial conducted by Bogenschutz et al. suggested that psilocybin promoted lasting abstinence in 10 individuals with alcohol use disorder as well^20^. Third, Pisano et al. conducted population-based survey research using The National Survey on Drug Use and Health and found that naturalistic classic psychedelic use was associated with lowered odds of opioid use disorder and related sub-criteria^21^. Fourth, a recent cross-sectional observational study conducted by Barbosa et al. found that rates of alcohol and tobacco use disorders were lower among individuals engaging in ceremonial use of ayahuasca—a South-American brew with psychoactive properties^22^. Most recently, online survey studies conducted by Garcia-Romeu et al. found naturalistic psychedelic use to be linked to reductions in cannabis, alcohol, stimulant, and opioid misuse^23,24^.

Although the research linking classic psychedelics to the alleviation of CUD is limited, existing research has linked the non-psychedelic hallucinogen ketamine to the alleviation of CUD. Dakwar et al. found ketamine infusions to reduce cocaine self-administration in a lab-based randomized cross-over trial^25^. Next, a 2019 randomized controlled trial found that a single ketamine infusion combined with a mindfulness relapse course diminished cocaine craving, promoted abstinence, and reduced relapse risk for CUD^26^.

Additionally, mediators of the link between ketamine and the alleviation of CUD suggest pathways through which classic psychedelics may plausibly protect against CUD. Research has found that mystical experience mediates the link between ketamine and improvements in CUD^27^; furthermore, research indicates that mystical experience may mediate the therapeutic effects of classic psychedelics as well^28,29^. Thus, although the link between ketamine and classic psychedelics as potential treatments or protective agents for CUD is purely suggestive, the aforementioned ketamine research indicates that classic psychedelics may represent a worthwhile frontier of exploration in order to identify possible protective associations with CUD.

Inspired by Pisano et al., we sought to test whether lifetime use of classic psychedelics is associated with lowered odds of CUD within the past year in nationally-representative population-based survey data. Additionally, informed by Jones and Nock (2022 [a, b, & c]), we sought to look at the potential protective associations that individual psychedelic substances share with CUD, as this prior research indicates that individual psychedelics share varying relationships to mental health and behavioral outcomes^30–32^.

While a population-based survey approach cannot be used to infer causality, the large, representative sample can allow for a robust preliminary inquiry into the link between various classic psychedelics and any protective associations with CUD.

## Method

Data for this project were from The National Survey on Drug Use and Health (NSDUH) (2015–2019) (unweighted *N* = 214,505), an annual survey that collects information on substance use and health outcomes within a nationally representative U.S sample ages 12 years and older. The NSDUH uses a computer-assisted self-interviewing format that involves NSDUH representatives administering the survey in participants’ homes. This study was exempt from review from the Harvard IRB as all data for this project are public (https://www.datafiles.samhsa.gov/), and all methods were carried out in accordance with relevant guidelines and regulations.

### Independent variables and covariates

Lifetime use (yes/no) of the following classic psychedelics served as our main independent variables: peyote, mescaline, psilocybin, LSD. We selected these substances as they are the most widely used classic psychedelics within the NSDUH survey. In keeping with previous population based survey research on hallucinogens and psychedelics^30–36^, we included the following demographic factors and lifetime substance use variables as covariates for our analyses: sex (male or female), age (18–25, 26–34, 35–49, 50 or older), race/ethnicity (Non-Hispanic White, Non-Hispanic Black, Non-Hispanic Native American/Alaska Native, Non-Hispanic Native Hawaiian/Pacific Islander, Non-Hispanic Asian, Non-Hispanic more than one race, or Hispanic), educational attainment (5th grade or less, 6th grade, 7th grade, 8th grade, 9th grade, 10th grade, 11th grade, 11th or 12th grade, high school diploma, some college (no degree), associate’s degree, or college degree or higher), self-reported engagement in risky behavior (never, seldom, sometimes, or always), annual household income (less than $20,000, $20,000–$49,999, $50,000–$74,999, $75,000 or more), marital status (married, divorced/separated, widowed, or never married), lifetime use of various substances (MDMA/ecstasy, heroin, PCP, inhalants, pain relievers, tranquilizers, stimulants, sedatives, and marijuana), and comorbid diagnosis of hallucinogen use disorder (based on DSM-IV criteria).

### Dependent variable

Our main dependent variables were overall past year CUD (dependence or abuse) and each of the 11 the criteria for CUD as defined by the *DSM-IV*.

### Analyses

We used multivariable logistic regression to test the associations between lifetime use of various classic psychedelics (peyote, psilocybin, mescaline, LSD) and CUD and its related *DSM-IV* criteria. Additionally, we used the ‘Survey’ package in R version 4.1.2 to incorporate the survey design of the NSDUH into our models^37^. The NSDUH uses a complex, sample-weighted survey design to ensure that responses are representative of U.S. population and do not over- or under-sample from particular demographic groups. Thus, incorporating the survey design into our analyses was essential to ensure that our study accurately captured any associations observed within the NSDUH data.

In our main model, we tested whether use of the four classic psychedelics was associated with lowered odds of CUD. If this first step of our analyses revealed any classic psychedelics to confer lowered odds of CUD, we would then conduct sensitivity analyses and test whether the substance(s) conferred lowered odds of the *DSM-IV* criteria for CUD, with lifetime use of all other substances and demographic factors serving as covariates. We used the DSM-IV criteria for CUD as outcome variables to align our approach with other population-based research on the link between psychedelics and substance use disorders^21^. Additionally, this approach utilizes virtually all CUD-related outcome variables in the NSDUH and allows for a more granular view of the protective associations that psychedelics may share with CUD.

## Results

### Preliminary analyses

The demographics of our sample, divided by those who do not meet criteria for CUD versus those who do (*N* = 1017) are presented in Table 1. Individuals meeting criteria for CUD are more likely than those without past year CUD to meet the following demographic profiles: single, less formally educated, younger, male, Black, lower-income, and more prone to risky behavior. Additionally, individuals with CUD were significantly more likely than those without CUD to have tried a classic psychedelic substance (peyote, mescaline, psilocybin, or LSD) and to meet criteria for comorbid hallucinogen use disorder.

**Table 1:** Demographic characteristics for those who do versus do not have cocaine use disorder (CUD).

Characteristic	Does not have CUD Unweighted N + (weighted %)	Has CUD Unweighted N + (weighted %)	*p* value^1^	
**Marital status**				< 0.001
Married	87,971 (52%)	109 (15%)		
Widowed	6,675 (5.9%)	22 (2.4%)		
Divorced or separated	22,982 (14%)	150 (20%)		
Never been married	95,860 (28%)	736 (63%)		
**Education**				< 0.001
Less than high school	27,670 (13%)	185 (19%)		
High school diploma/GED	56,735 (25%)	323 (30%)		
Some college credit	51,826 (22%)	295 (27%)		
College Degree or Higher	77,257 (41%)	214 (25%)		
**Age**				< 0.001
18–25	69,466 (14%)	450 (24%)		
26–34	43,752 (16%)	264 (29%)		
35–49	56,356 (25%)	210 (22%)		
50 +	43,914 (46%)	93 (24%)		
**Sex**				< 0.001
Male	99,154 (48%)	615 (67%)		
Female	114,334 (52%)	402 (33%)		
**Race/ethnicity**				< 0.001
Non-Hispanic White	128,323 (64%)	601 (60%)		
Non-Hispanic Black	26,934 (12%)	147 (20%)		
Non-Hispanic Native American/Alaska Native	3,059 (0.6%)	16 (0.7%)		
Non-Hispanic Native Hawaiian/Pacific Islander	1,086 (0.4%)	8 (0.4%)		
Non-Hispanic Asian	10,262 (5.6%)	13 (1.6%)		
Non-Hispanic more than one race	6,984 (1.7%)	57 (2.3%)		
Hispanic	36,840 (16%)	175 (14%)		
**Yearly household income**				< 0.001
< $20,000	42,586 (16%)	348 (32%)		
$20,000–$49,999	66,598 (29%)	348 (36%)		
$50,000–$74,999	33,392 (16%)	123 (12%)		
$75,000 +	70,912 (38%)	198 (20%)		
**Self-reported engagement in risky behavior**				< 0.001
Never	105,735 (55%)	176 (18%)		
Seldom	72,467 (32%)	301 (32%)		
Sometimes	30,304 (11%)	421 (41%)		
Always	4048 (1.3%)	116 (8.6%)		
Lifetime crack use	7024 (3.5%)	491 (56%)		< 0.001
Lifetime classic psychedelic use	30,050 (14%)	654 (66%)		< 0.001
Hallucinogen use disorder	276 (< 0.1%)	99 (8.1%)		< 0.001

^1^Chi-squared test with Rao & Scott's second-order correction.

### Associations between psychedelics and CUD

The results from our main study model, with lifetime use of various substances predicting past year CUD, are presented in Table 2, along with the frequency of use of each substance. Peyote was the sole substance to confer lowered odds of past year CUD, reducing odds of CUD by greater than 50% (aOR: 0.47). All other substances included in our model either had no association with CUD or were associated with increased odds of CUD.

**Table 2:** Results of multivariable logistic regression model predicting past year cocaine use disorder (CUD) + frequency of lifetime use of various substances (unweighted *N)* (demographic factors included as covariates).

Lifetime use	Frequency (unweighted N)	aOR (95% CI)^1^
**Classic psychedelics**		
Peyote	3766	0.47* (0.25, 0.89)
Mescaline	4595	1.10 (0.74, 1.62)
Psilocybin	22,276	1.26 (0.88, 1.78)
LSD	22,552	1.40* (1.00, 1.95)
**Other substances**		
MDMA/Ecstasy	21,195	2.61*** (1.90, 3.59)
PCP	3935	1.38 (0.93, 2.06)
Heroin	4790	3.20*** (2.28, 4.48)
Inhalants	21,856	1.29 (0.96, 1.74)
Pain Relievers	132,643	1.24 (0.91, 1.69)
Tranquilizers	48,572	1.80** (1.32, 2.44)
Stimulants	32,033	1.62** (1.19, 2.22)
Sedatives	27,218	0.84 (0.63, 1.13)
Marijuana	110,175	8.65*** (4.51, 16.6)

^1^*p < 0.05; **p < 0.01; ***p < 0.001; aOR = adjusted odds ratio; CI = confidence interval.

Significant values that indicate lowered odds of CUD are in bold.

### Sensitivity analyses

Given that peyote was the sole substance associated with lowered odds of CUD, we subsequently examined the relationships that peyote use shared with each of the 11 *DSM-IV* criteria for CUD, with lifetime use of all other substances and demographic factors serving as covariates. The results for the sensitivity analyses of peyote use predicting each of the 11 CUD criteria, as well as the frequency of each CUD criterion, are present in Table 3. Overall, use of peyote was associated with lowered odds of the majority (seven of 11) of the CUD criteria; additionally, these associations were strong as peyote reduced odds of each of the criteria by more than 50%.

**Table 3:** Sensitivity analyses—results from eleven multivariable logistic regression models assessing the associations of peyote use (independent variable) to DSM-IV criteria for cocaine use disorder (CUD) (lifetime use of all other substances and all demographic factors included as covariates) + criteria frequency.

Cocaine use disorder criteria	Frequency (unweighted N)^1^	aOR (95% CI)^1^
**Dependence criteria**		
1. Significant Time Spent Getting/Using	982	0.44* (0.22, 0.90)
2. Use More Than Intended	445	0.32* (0.13, 0.82)
3. Decreased Effects/Need More for Same Effect	1,041	0.47* (0.23, 0.95)
4. Unable to Cut Back	310	0.26* (0.09, 0.78)
5. Emotional/Physical Health Problems	561	0.53 (0.24, 1.16)
6. Fewer Important Activities	580	0.55 (0.25, 1.21)
7. Feeling Blue & 2 + Withdrawal Symptoms	434	0.65 (0.30, 1.42)
**Abuse criteria**		
8. Significant Work/Home/School Problems	505	0.42* (0.18, 0.96)
9. Use in Physically Hazardous Situations	518	0.36** (0.18, 0.69)
10. Legal Trouble	223	0.47 (0.14, 1.53)
11. Relational Issues	377	0.32* (0.11, 0.90)

Significant values that indicate lowered odds of CUD criteria are in bold.

^1^*p < 0.05; **p < 0.01; ***p < 0.001; aOR = adjusted odds ratio; CI = confidence interval.

### Post-hoc analyses of demographic differences between peyote users versus peyote + cocaine users

Given the previously described demographic differences between individuals who do versus do not meet criteria for CUD, we also conducted post-hoc chi-squared analyses to explore any demographic differences for lifetime peyote users who have versus have not used cocaine. If amongst peyote users one sees a significantly different sub-population of individuals that consume cocaine and thus are at risk for CUD, these findings would suggest third-variable demographic factors contribute to our findings. Results for these analyses are reported in Table 4. Overall, these results revealed significant differences between the two groups based on the following demographic factors: marital status, education level, age, sex, race. There were no differences in yearly household income.

**Table 4:** Demographics of peyote users who have versus have not used cocaine.

Characteristic	Lifetime Peyote Use Only Unweighted N + (weighted %)	Lifetime Peyote + Cocaine Use unweighted N + (weighted %)	*p* value^1^
**Marital status**			0.044
Married	380 (49%)	1170 (47%)	
Widowed	41 (7.1%)	106 (4.1%)	
Divorced or Separated	154 (24%)	692 (26%)	
Never Been Married	332 (21%)	891 (23%)	
**Education**			0.015
Less than High School	123 (10%)	244 (7.2%)	
High school diploma/GED	226 (20%)	760 (23%)	
Some college credit	227 (22%)	827 (28%)	
College Degree or Higher	331 (47%)	1028 (42%)	
**Age**			< 0.001
18–25	190 (6.0%)	294 (3.0%)	
26–34	148 (9.8%)	422 (8.6%)	
35–49	279 (23%)	773 (16%)	
50 +	290 (61%)	1370 (72%)	
**Sex**			0.005
Male	566 (66%)	2062 (73%)	
Female	341 (34%)	797 (27%)	
**Race/ethnicity**			< 0.001
Non-Hispanic White	542 (76%)	2246 (85%)	
Non-Hispanic Black	22 (2.0%)	61 (1.9%)	
Non-Hispanic Native American/Alaska Native	182 (8.4%)	102 (1.0%)	
Non-Hispanic Native Hawaiian/Pacific Islander	6 (0.2%)	11 (0.1%)	
Non-Hispanic Asian	7 (1.2%)	21 (0.7%)	
Non-Hispanic more than one race	56 (2.9%)	160 (3.4%)	
Hispanic	92 (9.7%)	258 (7.7%)	
**Yearly household income**			0.8
< $20,000	215 (17%)	566 (17%)	
$20,000-$49,999	292 (30%)	917 (29%)	
$50,000-$74,999	129 (16%)	440 (15%)	
$75,000 +	271 (36%)	936 (39%)	
Lifetime Crack Use	0 (0%)	978 (31%)	< 0.001

^1^Chi-squared test with Rao & Scott's second-order correction.

## Discussion

The goal of this paper was to assess whether lifetime use of four commonly used classic psychedelics (peyote, psilocybin, mescaline, LSD) shared protective associations with past year CUD. Overall, peyote was the sole substance associated with lowered odds of CUD, with every other substance (including other classic psychedelics) either sharing no association or conferring increased odds of CUD. Furthermore, to confirm that the association between peyote use and lowered odds of CUD was not spurious, we conducted sensitivity analyses and found that lifetime peyote use was associated with lowered odds of the majority of CUD criteria.

### Limitations

There are many limitations to this work that are important to state clearly. First and foremost, the associations reported in this study are correlational and cannot be used to infer causality. There may be indirect or third-variable factors that link peyote use to decreased odds of CUD. Future longitudinal studies can shed further light on whether peyote can effectively act as a protective agent for CUD.

Second, given that peyote use was assessed over a lifetime and CUD was assessed over the past year, we cannot establish clear temporal precedent between peyote use and CUD. Furthermore, our lifetime use variable does not allow us to assess recency or frequency of use as well. However, given that classic psychedelics can elicit protective effects over multiple years after just a few uses^38^, it remains plausible that peyote works as a causal agent to reduce CUD.

Third, there are important limitations to the demographics of our sample as the NSDUH does not collect data from key populations that may be essential for the study of substance abuse. The NSDUH does not survey anyone experiencing homelessness, currently incarcerated individuals, or active-duty military members. These populations are particularly important to study given the potentially elevated rates of CUD that may exist within these groups. Future studies that examine the link between psychedelic use and lowered odds of CUD in these populations are crucial to better understand our observed findings related to peyote.

#### Potential harm associated with LSD and classic psychedelic use

Lastly, it is possible that harm from peyote or other classic psychedelics occurred on the individual or group level. The finding that LSD was associated with increased odds of CUD lends particular credence to this possibility and accords with other population-based research that links LSD to increased odds of adverse outcomes^31^. Below, we will provide a more in-depth reflection on this limitation and discuss the potential pathways by which classic psychedelics may lead to adverse outcomes and increased odds of CUD.

*“Bad trips.”* First, acute classic psychedelic use can cause experiences of paranoia, anxiety, and extreme distress during “bad trips”^39^. As negative affect is linked to relapse and the exacerbation of substance use disorders^40,41^, these acute adverse experiences may explain why LSD and/or other psychedelics in some instances may lead to increased odds of CUD.

*Hallucinogen use disorder.* Second, psychedelics can cause hallucinogen use disorder, causing one to abuse these substances such that they put the user or others at risk or cause significant issues at work, at school, or within interpersonal relationships^42,43^. Given the significant co-morbidity between hallucinogen use disorder and CUD observed within this sample, misuse of classic psychedelics may drive or exacerbate the misuse of cocaine.

*Increased risk for psychosis.* Third, psychedelics have been linked to increased risk of psychosis^39,44–46^; however, much of the evidence supporting this link is historical. Nevertheless, there is a well-established link between psychotic disorders and substance use issues^47–50^. Thus, LSD may increase risk for CUD by increasing risk for psychotic disorders. However, given the limitations to the research on the link between psychedelics and risk for psychosis, more research is needed to establish this potential risk pathway.

*Hallucinogen persisting perception disorder.* Finally, in rare instances, classic psychedelics have been linked to lasting perceptual abnormalities in a condition referred to as hallucinogen persisting perception disorder (HPPD). This condition has been linked to LSD use and is reported to cause significant morbidity^51^. However, the research on HPPD is scant. Thus, future research can elucidate whether the morbidity associated with HPPD might lead to increased risk of substance use disorders and CUD.

Overall, the differing results between peyote and LSD indicate that classic psychedelics share complex and nuanced relationships with mental health outcomes at the population level. Better understanding moderating factors of classic psychedelic use with mental health outcomes can shed light on potential harm that may be occurring as a result of psychedelic use.

### Potential mediators of the association between peyote use and decreased odds of CUD

Despite the above limitations, our study makes an important contribution to the literature and establishes a preliminary link between peyote use and lowered odds of CUD. Furthermore, this study is one of the first within Western science to link peyote use to lowered odds of deleterious outcomes as well, as just a handful of studies exist that link peyote or its constituent compounds (mescaline) to salutary mental health and behavioral outcomes^32,52,53^. This link lays the foundation for future work that investigates peyote as either a treatment for CUD or a protective agent that lowers the likelihood of developing CUD. Overall, our findings accord with a large body of population-based research linking naturalistic classic psychedelic use to lowered odds of deleterious health and behavioral outcomes^31,32,34,35,54–56^.

Additionally, future research should examine the pharmacological dynamics of peyote, as further investigation may surface causal mediators underlying the link between peyote use and lowered odds of CUD. Additionally, these investigations can shed light on a key question raised by our study: why mescaline and peyote shared differing associations to CUD. Given that mescaline is the primary psychoactive compound in peyote, one might reasonably expect both compounds to confer lowered odds of CUD. However, peyote consists of a blend of many different psychoactive and non-psychoactive alkaloids, which may lead to differing pharmacological effects resulting from peyote use compared to mescaline use^57^. Better understanding the chemical composition of peyote may elucidate the observed differences between peyote and mescaline.

#### Third variable/demographic factors

In addition to better understanding the pharmacological dynamics of peyote, there is a need to also better understand indirect/third-variable factors and potential population differences between individuals who use peyote and the broader population, as these factors may underlie our observed findings as well.

As has been named in previous population-based research on classic psychedelics, pre-drug personal factors like higher levels of the personality trait openness and higher levels of spirituality may be simultaneously associated with higher rates of classic psychedelic use and lowered odds of deleterious mental health outcomes^31,54^. For instance, a 2006 study by ter Bogt et al. found that there were personality differences between individuals who did versus did not consume MDMA in a naturalistic setting^58^. Additionally, another study conducted by Nour et al. found lifetime psychedelic use (but not lifetime cocaine use or alcohol consumption patterns) to be associated with liberal political views and openness^59^. More recent research has also found potential pre-drug differences associated with psychedelic use. Erritzoe et al. found psychedelic use to be associated with openness in a cross-sectional study featuring 25 hallucinogen users, and Johnstad (2021) found psychedelic use to be linked to higher scores on each of the Big Five personality traits except for extraversion (agreeableness, openness, conscientiousness, and neuroticism)^60,61^. Thus, evidence indicates that pre-existing personality factors may indeed contribute to our observed associations between peyote and lowered odds of CUD.

Demographic differences likely underlie our observed findings as well. Our analyses comparing individuals with CUD versus without CUD, as well as those comparing peyote users who have versus have not used cocaine, revealed significant differences across the dimensions of race, education, marital status, and educational attainment. Thus, these differences likely contributed to our results. Although we controlled for these factors in conducting our analyses, there are likely demographic factors associated with these traits that we could not control for due to limitations inherent to the NSDUH dataset. For instance, as noted above, the NSDUH does not collect data on or sample from individuals experiencing homelessness, currently incarcerated individuals, or individuals who are active-duty military members; accordingly, there are likely many additional demographics not included in the NSDUH that could shed further light on our observed findings on peyote and CUD. In addition, studies on the epidemiology of cocaine use and CUD also suggest complex interactions between demographic factors and disordered cocaine use^62,63^. Thus, future analyses should more thoroughly investigate how identity factors contribute to and/or moderate our observed associations.

#### Contexts for peyote use

Additionally, further studies into motivations and common contexts for peyote use can produce invaluable information on the link between peyote and CUD as well. “Set” (the mindset of an individual taking psychedelics) and the “setting” within which one consumes a psychedelic substance markedly shape the psychedelic experience^64^. Thus, better understanding general contexts for peyote use are critical to understanding exactly why peyote confers lowered odds of CUD. This line of inquiry can also potentially shed light on the observed differences in findings between peyote and mescaline: if individuals seek out these substances for different reasons, and take them in different contexts, it could explain the differing odds observed for these two closely related substances.

## Conclusion

CUD is a major public health issue for which there are few effective behavioral or pharmacological treatments. Furthermore, risk factors and protective factors for CUD remain poorly understood. This study demonstrates that naturalistic lifetime peyote use is associated with lowered odds of CUD and a majority of CUD criteria. Future longitudinal studies investigating the link between peyote and CUD, as well as future investigations into the genetic and behavioral profiles of individuals who use peyote, can shed further light on potential treatments and protective factors for CUD. Additionally, future studies should also investigate how classic psychedelics and LSD in particular may lead to increased risk for CUD. Overall, this study represents incremental progress towards better understanding, treating, and preventing CUD.

## Data Availability

The data supporting the findings from this project are publicly available at the Substance Abuse & Mental Health Data Archive (SAMHDA) at the following web address: https://www.datafiles.samhsa.gov/.
